# Knowledge, attitude and use of evidence based practice (EBP) among registered nurse-midwives practicing in central hospitals in Malawi: a cross-sectional survey

**DOI:** 10.1186/s12912-022-00916-z

**Published:** 2022-06-06

**Authors:** Paul Uchizi Kaseka, Balwani Chingatichifwe Mbakaya

**Affiliations:** 1 Paediatric Department, Mzuzu Central Hospital, Private Bag 209, Mzuzu, Malawi; 2 Faculty of Applied Sciences, Department of Public Health, University of Livingstonia, Mzuzu, Malawi; 3 Faculty of Health Sciences, Mzuzu University, Mzuzu, Malawi

**Keywords:** Knowledge, Attitude, Evidence-based practice, Use, Registered Nurse-midwives

## Abstract

**Background:**

Even though evidence based practice (EBP) is being considered as a critical element in improving the quality of health services and achieving excellence in patient care, there is currently little knowledge of how EBP relates to nursing and midwifery in Malawi. This paper is a report of a study describing EBP knowledge, attitudes, and use of registered nurse-midwives practicing in central hospitals across Malawi.

**Methods:**

The descriptive, cross-sectional research design was conducted with a randomly selected sample of 183 nurse-midwives (response rate of 87.9%). The study used a paper version questionnaire to collect the data. The data were analysed using both descriptive and inferential statistics in the Statistical Product and Service Solutions version 23. Descriptive statistics were calculated to summarise overall knowledge levels, attitudes, and use of nurse-midwives as percentages based on their scores on the assessment scale (1 to 7 Likert scale) in the EBP questionnaire. Non-parametric Mann-Whitney and Kruskal-Wallis tests were carried out to compare evidence-based practice scores based on demographics. Pearson’s correlation (r) and stepwise regression analysis were further performed to analyse the relationship between the knowledge, attitude and use of nurse-midwives on the overall EBP of nurse-midwives.

**Results:**

The average scores (mean±SD) of evidence-based practice amongst nurse-midwives were 78.7 ± 19.6 for attitude, 70.6 ± 15.1 for knowledge levels, 57.8 ± 23 for use, and 68.9 ± 14.2 for the overall EBP. Higher educational qualification was associated with higher scores in knowledge levels (*P* = 0.02). Research experience was associated with higher scores in nursing use (*P* = 0.005), and higher overall evidence-based practice were associated with both research experience (*P* = 0.035) and educational qualification (*P* = 0.004). Nurse-midwives attitude was affected by clinical experience (*P* = 0.006) and the hospital where nurse-midwives worked (*P* = 0.016). There was no significant difference in the EBP scores of nurse-midwives based on gender and/or their administrative roles in their respective central hospitals.

**Conclusion:**

It is important to develop the knowledge or skills of nurse midwives in order to enhance evidence-based practice amongst nurse-midwives in Malawian hospitals. The results can be used by nurse managers, nurse educators, policy makers at the Ministry of Health and Nurses and Midwives Council of Malawi to enhance implementation of EBP.

## Background

Evidence-based practice (EBP) is now being recognised by healthcare policy makers, healthcare staff, researchers and regulatory agencies, as the gold standard for the provision of safe and compassionate healthcare [1, 2]. EBP is being considered as a critical element in improving the quality of health services and achieving excellence in patient care [3]. EBP has been described as a decision-making process for patient care through integration of knowledge provided by the best available evidence, with a practitioner’s clinical experience and judgement, and combined with patient’s own values and preferences [4]. Registered nurse-midwives have a key role to ensure that high-quality and consistent services are provided to their clients [5]. The international Council of Nurses (ICN) has demonstrated a commitment to nurses being active in developing a core of research-based professional knowledge that supports evidence-based practice (EBP) [6].

Previous studies have suggested that incorporating research findings into clinical practice brings about a higher level of nursing care as well as improved patient outcome, increased patient satisfaction, reduced hospital stay, efficient use of resources, elimination of unnecessary practices and improved cost efficiency [7–9]. For example, in a study by Hansen and Severinsson [9], using EBP enabled nurses to remain up-to-date with healthcare trends and improved the care by promoting the use of the most appropriate and effective interventions which led to improved outcomes for the patients, nurses and hospitals. Moreover, nurses who are involved in EBP have been found to raise their status in multi-professional teams, and express a sense of professionalism and growth which contributes to their professional identity [10].

While EBP is rapidly progressing in many developed countries like Ireland, Colombia, Chile, Spain, United States of America, and United Kingdom [11–14], several published studies in Africa just indicate that EBP in nursing and midwifery is at its infancy stage [15–18], where requests for the provision of evidence does not come from the practice sites [19] as most of the evidence is in form of secondary evidence that comes as clinical guidelines. In some African countries the EBP utilisation is below 20%. For example, in Ethiopia only 15.7% of nurses use research evidence in clinical practice, while in Kenya only 8% utilise research evidence when providing nursing and midwifery care [15, 18].

The need for evidence in healthcare decision has significantly increased in low and middle income countries due to high burden of diseases and illnesses. According to Oxman, Lavis, Lewin and Fretheim [20], countries in the sub-Saharan region of Africa can multiply on their efforts to improve the health outcomes in their countries if their measures in the prevention of death and disability considers the local context, and that the choice of these interventions and policies are based on strong scientific evidence. This agrees with the recommendations by the World Health Organisation (WHO) that health and social services in general and nursing practice in particular should be based on the best research evidence [21]. According to Vorthems, Spoden and Wilcken [22], implementing an evidence-based oncology outpatient staffing system based on patients’ acuity improved staff efficiency, reduced workload, and improved patient and staff satisfaction within a 6 month period. Similarly, Muhumuza, Gomersall, Fredrick et al. [23] through their improved health care worker hand hygiene project in Uganda, observed that evidence based practice can be used as a tool to improve health practice in a low-resource setting.

In the recent systematic review by Lizarondo, Lockwood and McArthur [24] it was observed that even though EBP demonstrated to positively impact patient outcomes, however, nurses and midwives in Africa had difficulty in incorporating it into their practice. This collaborates with other studies that have also proved that nursing is still not based on best available evidence [1, 16, 25–29]. The consistently reported barriers include lack of knowledge and skills to evaluate research findings, insufficient time, the right to change practice, lack of administrative support, organisational cultures rewarding routine task-based practice, poor attitude towards EBP, lack of value for research in practice, difficulties to comprehend the databases, difficulties in searching research materials, and lack of knowledge to access and navigate through nursing databases, poor understanding of statistics and critical appraisal, lack of knowledge among nurses without a university education, and heavy workload [28, 30, 31].

Van Achterberg, Schoonhoven and Grol [32] point out that nurses are not a homogenous target group, but are professionals with varying educational levels, specialisations, work settings, and patient populations which they serve. These differences are potentially relevant to determine knowledge, attitudes and practices. According to Ammouri, Raddaha, Dsouza, et al. [30] approaches to encourage the adoption of EBP among nurse-midwives should be based on evidence which addresses the current barriers to the adoption of EBP in the clinical setting. This is what motivated the researchers to explore knowledge, attitude, and use of EBP among registered nurse-midwives (RNMs) practising in central hospitals in Malawi. The study was conducted to add knowledge to the nursing and midwifery professions by establishing an empirical foundation for the current knowledge, attitudes, and practices of RNMs on EBP. This helped in facilitating appropriate strategies needed to help RNMs across the healthcare delivery system to utilise evidence in their practices. It is expected that findings from this study will help come up with recommendations for appropriate education and clinical programs to ensure successful implementation of EBP in Malawian hospitals. This will result in improved and cost effective patient care in Malawi which is a low income country.

### Purpose and AIMS

This study was conducted to assess how EBP was perceived and used by the RNMs practising in central hospitals across Malawi. Specifically, this study aimed to address the following research objectives:

1. to determine the EBP knowledge or skills, attitudes and practices of RNM practicing in central hospitals in Malawi.
2. to explore if there were any differences between knowledge or skills, attitudes, and practice among clinical RNMs based on their education level.
3. to determine if there were any differences between knowledge or skills, attitudes, and practice among clinical RNMs based on their gender.
4. to determine if there were any differences between length of work experience and RNMs knowledge or skill, attitudes, and practice of EBP.

The study research questions were

1. What are the EBP knowledge or skills, attitudes and practices of RNM practicing in central hospitals in Malawi?
2. Are there any differences between knowledge or skills, attitudes, and practice among clinical RNMs based on their education level?
3. Are there any differences between knowledge or skills, attitudes, and practice among clinical RNMs based on their gender?
4. Are there were any differences between length of work experience and RNMs knowledge or skill, attitudes, and practice of EBP?

## Materials and methods

### Study design

This study used a descriptive, cross sectional design to investigate knowledge, attitude, and use of EBP among RNMs practicing in central hospitals across Malawi from January to March 2019.

### Study setting

The study was conducted in all four central hospitals in Malawi. Central hospitals in Malawi are tertiary level institutions that provide specialist care [33]. The study was conducted in these central hospitals because they serve as referral centres for all other health facilities in all the three regions of Malawi (northern, central and southern regions). The central hospitals have the majority of most RNMs.

### Population and sampling

The study population consisted of the RNMs from all the units in the four central hospitals. According to the statistics obtained from the central hospitals, in October 2018 there were 326 permanently employed RNMs registered with Nurses and Midwives Council of Malawi (NMCM), and practising in all the settings of the central hospitals in Malawi. There were 120 RNMs at Queen Elizabeth Central Hospital (QECH), 107 RNMs at Kamuzu Central Hospital (KCH), 55 RNMs at Mzuzu Central Hospital (MCH) and 44 RNMs at Zomba Central Hospital (ZCH). The total population for this study was therefore 326 RNMs with a diploma and above as their highest qualifications.

A sample size of 208 was found adequate based on sample size calculation using Open-Epi sample size calculator.

$$\mathrm{Sample}\ \mathrm{size}\ \boldsymbol{n}=\left[{\mathbf{DEFF}}^{\ast}\mathbf{Np}\left(\mathbf{1}-\mathbf{p}\right)\right]/\left[\right({\mathbf{d}}^{\mathbf{2}}/{{{\mathbf{Z}}^{\mathbf{2}}}_{\mathbf{1}-\boldsymbol{\upalpha} /\mathbf{2}}}^{\ast}\left(\mathbf{N}-\mathbf{1}\right)+{\mathbf{p}}^{\ast}\left(\mathbf{1}-\mathbf{p}\right)\Big]$$

Population size for finite population correction factor (N): 326.

Hypothesized % frequency of outcome factor in the population (p): 50%+/− 5.

Confidence limit as of 100 (absolute +/−%)(d): 5%.

Design effect (for cluster survey-DEFF):1.

Confidence level (%): 95%.

Sample size = 177.

Response rate of 85% = 208.

In this study a stratified random sampling was used to maximise representativeness. First, all central hospitals were classified based on size (large or small). The size of the central hospital in this study was based on current number of RNMs working at each hospital. From the four hospitals, participants working within them were selected, stratified by workplace size and field of study (nursing and midwifery). The resulting sample consisted of 208 individuals with all central hospitals proportionately represented relative to the size of the stratum in the population. A total number of 208 questionnaires were distributed and 183 were returned resulting in 87.9% response rate. The response rate was sufficiently high that the results are considered generalizable to the hospital setting where participants were recruited.

### Data collection instrument

A paper version of a self-administered Evidence-Based Practice Questionnaire (EBPQ) was used to explore respondents’ practice, knowledge, attitude and use associated with EBP. This data collection tool was developed by Upton and Upton [34] in the United Kingdom to assess the EBP knowledge, attitudes, and practice among nurses. This instrument identified through literature review and previously published studies [2, 17, 30] is a self-report scale which explores the day-to-day use of EBP.

This EBPQ is a self-completion questionnaire, composed of twenty-four items distributed among three subscales: knowledge or skills (14 items), attitudes (4 items), and use (practice) (6 items). Each item is scored on a scale of one to seven, with a higher score being associated with a more positive attitude towards EBP or use and knowledge of EBP, and a lower score associated with negative attitude or use and knowledge of EBP. Responses of each item were considered positive if scores were greater than 4 [30]. This instrument consists of three sub-scales: practice, knowledge or skills, and attitudes.

Previous studies have shown that this instrument is valid and reliable. The original study done to check the validity and reliability of the instrument demonstrated a Cronbach’s alpha of 0.87 for the entire questionnaire, 0.85 for the practice of EBP sub-scale, 0.79 for the attitudes sub-scale, and 0.91 for the knowledge or skills sub-scale [34]. Construct validity was established using an independent EBP measure yielding a moderately positive relationship between scales [34]. A study done by AbuRuz, Hayeah, Al-Dweik and Al-Akash [3] supported the reliability of this instrument as the following: the Cronbach’s alpha coefficient of the EBPQ was 0.96 for the entire questionnaire, 0.93 for the practice of EBP sub-scale, 0.82 for the attitudes sub-scale, and 0.95 for the knowledge or skills sub-scale. In this study, the Cronbach’s alpha coefficient was 0.85 for the entire questionnaire, 0.83 for the practice subscale. 0.79 for the attitude subscale and 0.87 for the knowledge/skill subscale. For data analysis, we applied both means of total and average EBPQ scores to report each subscale, in order to perform comparison with similar studies.

The tool’s demographic data was slightly modified to collect more data. Information on nationality, age, sex, highest level of education, workplace, and years of nursing experience, nursing position/grade, and an open-ended question to elicit information related to EBP not covered in the questionnaires or respondents’ opinions were collected.

### Data collection procedure

RNMs recruited in this study were those that were practicing in the central hospitals, and certified for clinical practice by NMCM regardless of their professional qualification. Stratified random sampling was used to maximise representativeness. First, all central hospitals were classified based on size (large or small). The lists of all RNMs were requested from nurse managers of the central hospitals. Study participants were selected randomly, with the number of RNMs invited to participate from each setting proportional to the total number of RNMs working in their respective facilities. A list of all RNMs who were invited to take part in the study for each central hospital was created and these RNMs were then visited in their workplaces by the research assistants. RNMs of any gender and cultural background or ethnicity who agreed to participate in the study and met the following inclusion criteria were included: 1) holding at least a university diploma in nursing and above; 2) employed permanently in the selected facilities; 3) have at least 1 year of clinical experience; 4) signed an informed consent. Nurse-midwife technicians, enrolled nurse midwives, auxiliary nurses, and community midwife assistants were not included in the study as these categories of nursing or midwifery staffs are not expected to have knowledge and skills of EBP based on the training syllabi and curriculum which they undergo during their training. All qualified RNMs from all units and all shifts were included, but RNMs who were not permanently employed in the facilities were excluded. Data collection took place between January and March 2019.

### Data analysis

All collected data were analysed using both descriptive and inferential statistics in the Statistical Product and Service Solutions (SPSS) version 23. Descriptive statistics were calculated to summarise overall knowledge levels, attitudes and practices of nurse midwives as percentages based on their scores from the assessment scale (1 to 7 Likert scale) in the EBP questionnaire. Non-parametric Mann-Whitney and Kruskal-Wallis tests were carried out to compare EBP scores based on demographics. Pearson’s correlation (r) and stepwise regression analysis were further carried out to analyse the relationship between the knowledge, attitudes and practices of nurse-midwives on the overall EBP of nurse-midwives.

Stepwise regression was used to identify a useful subset of the predictors from the list of variables. It involved a step-by-step iterative construction of a regression model that involved the selection of independent variables to be used in a final model. EBP of nurse-midwives was the response variable in this study. The explanatory variables that were not significantly correlated to EBP of nurse-midwives were removed in succession and testing for statistical significance using Pearson correlation was done after each iteration. The explanatory variables that were significantly correlated with the response variable during the iteration process were then together used to build a binary logistic regression model to generate predictors of EBP of nurse-midwives.

### Validity and reliability

To ensure validity and reliability, a sample size calculation ensured that a substantial high number of RNMs were recruited for generalizability of the study. The study questionnaire was pretested to ensure that questions used in this study were clear. The EBPQ was modified for easy understanding of participants. Cronbach Alpha was also calculated for the modified EBPQ and was found to be fairly adequate.

## Results

### Demographic characteristics

Out of 208 questionnaires distributed, 183 questionnaires were returned (response rate 87.9%). Among the participants, 182 were Malawians and one was a Filipino. The majority of participants (79.2%) were females, with 20.8% males. Forty-seven percent of the participants were aged between 20 and 29 with 5.5% aged between 50 and 59 years. The majority (78.7%) had a bachelors’ degree as their highest qualification, with 14.2% having a master’s degree, and 6.6% having university nursing diploma.

Of all the participants, 59.6% had less than 5 years of practical experience, 33.3% had five to 10 years of practical experience and 6.1% had more than 10 years of practical experience. The demographic characteristics are highlighted in Table 1.

**Table 1:** Participants’ demographic characteristics (*n* = 183)

	***Frequency***	***Percentage***
***Gender***		
Female	*145*	***79.2***
Male	*37*	***20.8***
**Total**	***183***	***100***
***Age (years)***		
20–29	***87***	***47.5***
30–39	***65***	***35.5***
40–49	***21***	***11.5***
50–59	***10***	***5.5***
***Total***	***183***	***100***
***Highest professional qualification obtained***		
Diploma in nursing	*12*	***6.6***
Bachelor’s Degree in nursing and midwifery	*144*	***78.7***
Master’s Degree in nursing or midwifery	*26*	***14.2***
	***182***	***99.5***
***Current position and Grade***		
Nursing sister (Diploma)	*12*	***6.6***
Nursing and Midwifery Officer (I)	*144*	***78.7***
Senior Nursing and Midwifery Officer (H)	*15*	***8.2***
Principal Nursing and Midwifery Officer (G)	*5*	***2.7***
Chief Nursing and Midwifery Officer (F)	*4*	***2.1***
Deputy Hospital Director (Nursing and Midwifery) (E)	*3*	***1.6***
**Total**	***181***	***99.9***
***Place of work***		
QECH	64	35.0
KCH	61	33.3
MZCH	31	16.9
ZCH	27	14.8
**Total**	**183**	**100**
**Practical (clinical) experience**		
< 5 years	109	59.6
5–10 years	63	33.3
> 10 years	11	6.1
**Total**	181	99.0
**Total**	**183**	**100**

### Participants’ knowledge, attitudes and practices of EBP

In this study, participants self-rated their knowledge or skills, attitude and practice on a 7 point Likert scale, where 1 represented poor and 7 represented excellent. The items’ scores of 1 to 4 were combined and represented as negative items and the scores of 5 to 7 were combined and represented as positive items. The average scores of the total EBPQ ranged from 3.56 ± 1.9 to 6.16 ± 2.9, with a mean of 4.86. The subscale of attitude was the highest one with mean of (4.85 ± 1.92 to 6.16 ± 2.9), followed by that of knowledge (4.34 ± 1.53 to 5.46 ± 1.49), and the lowest one was practice (3.56 ± 1.9 to 3.85 ± 1.7) (Table 2).

**Table 2:** Scores of each EBP questionnaire item from all respondents (*n* = 183)

ITEM	Score (mean ± SD)	Score (%)	Priority item rank
**Practice**			
Critically appraising literature	3.56 ± 1.9	50.9	1
Evaluating the outcomes of own practice	4.41 ± 1.96	63	2
Sharing information with colleagues	4.49 ± 1.95	64.17	3
Integrating evidence with expertise	4.14 ± 2	59.09	4
Finding relevant evidence	3.84 ± 1.9	54.8	5
Formulating clear questions	3.85 ± 1.7	55.04	6
**Attitudes**			
Making time in a work schedule for research	4.85 ± 1.92	69.16	1
Welcoming questions in own practice	5.65 ± 1.66	80.72	2
Changing practice due to evidence found	5.59 ± 1.58	79.94	3
EBP is fundamental to professional practice	6.16 ± 2.9	84.86	4
**Knowledge or skills**			
Research skills	4.34 ± 1.53	62.37	1
Critically analysing evidence against set standards	4.87 ± 1.52	69.71	2
Retrieving evidence	4.77 ± 1.65	68.23	3
Determining the validity (close to the truth) of material	4.93 ± 1.42	70.41	4
Converting information needs into research questions	4.11 ± 1.56	58.63	5
Awareness of major information types/sources	4.57 ± 1.44	65.11	6
Determining how useful (clinically applicable) material is	5.33 ± 1.43	76.11	7
IT skills	4.53 ± 1.43	64.72	8
Monitoring and reviewing practice	4.74 ± 1.51	67.68	9
Applying information to individual cases	5.55 ± 1.31	79.08	10
Identifying gaps in professional practice	5.4 ± 1.38	77.05	11
Reviewing own practices	5.57 ± 1.34	79.47	12
Disseminating new ideas about care to colleagues	5.28 ± 1.55	75.41	13
Sharing ideas and information with colleagues	5.46 ± 1.49	77.91	14

The lowest score and the most negative one for attitude was for the item “making time in a work schedule for research.” (Table 2). The lowest scores for knowledge were received in areas to do with converting information needs into research questions, research skills, critically analysing evidence against set standards and retrieving evidence. The highest mean score (5.46 ± 1.49) in knowledge was for sharing ideas and information with colleagues. The nurse midwives in this study scored relatively low in the entire practice on EBP section. The item with the lowest practice score was “critically appraising literature.” The respondents’ mean score for this item was 3.56 ± 1.9 out of 7 and it was also the most negative one in practice subscale, with 50.9% negative rate (Table 2).

### Comparisons of groups of registered nurse midwives

#### Relationships

Results on the effect of different demographics on the knowledge or skills, practice and attitude of nurse-midwives in Malawian central hospitals is presented in Table 3*.*

**Table 3:** Summary of comparisons for nursing practice, attitude, knowledge levels and total EBP scores (%) based on different nurse demographics

Characteristics	Nursing Practice	Nursing Attitude	Knowledge level	Total EBP
**Gender**				
Male	61.1	79.8	73.2	71.3
Female	57.4	78.9	70.8	68.8
*p*-value	0.497	0.593	0.355	0.357
**Clinical experience**				
< 5 years	60	82.2	71.6	70.5
5–10 years	55.6	76	71.7	68.4
> 10 years	53.7	64	64.4	61.6
*p*-value	0.321	0.006*	0.305	0.093
**Position/Nursing administrator**				
Yes	57.8	75.5	73.5	68.9
No	58.2	79.7	70.9	69.2
*p*-value	0.919	0.4	0.345	0.756
**Specialty/Research experience**				
Yes	60.8	80.5	74.5	73.4
No	55.9	78.7	70.3	68.1
*p*-value	0.013*	0.97	0.206	0.035*
**Education/Qualification**				
Diploma	42.5	72.9	65.6	61.1
Bachelors	57.8	79.3	70.5	68.8
Masters	67.5	80.8	78.4	75.9
*p*-value	0.005*	0.308	0.02*	0.004*
**Institution/Facility**				
MCH	54.8	78	72.2	68.8
KCH	58.9	73	71.4	68.6
QECH	59.7	83.2	71.5	70.5
ZCH	57	83.1	70.1	69
*p*-value	0.871	0.016*	0.995	0.583

NB: All scores for the different characteristics are percentages (%). Scores of the respondents within each category are significantly different where *p*-value < 0.05 is flagged by an asterisk (*)

The study revealed statistically significant differences in the nursing practice (*P* = 0.005), knowledge levels (*P* = 0.02) and total EBP (*P* = 0.004) based on educational qualification of the nurse-midwives. Higher scores were obtained in nurse-midwives in possession of Master’s degrees for nursing practice (67.5%), knowledge levels (78.4%) as well as total EBPQ (75.9%), in contrast to nurse-midwives with Diplomas and Bachelor’s degrees for nursing practice (42.5%; 57.8%), knowledge levels (65.6%; 70.5%) and total EBP (61.1%; 68.8%) respectively. However, the nursing attitude did not significantly differ amongst the nurse-midwives based on their educational qualifications (*P* = 0.308).

Furthermore, the results revealed no statistically significant differences in nursing practice, attitude, knowledge levels, and overall EBP based on gender (*P* > 0.05). Similarly, EBP amongst nurses and midwives was not influenced by administrative roles or positions held by nurse-midwives in hospitals (*P* > 0.05), even though significantly higher scores in nursing attitude (*P* = 0.016) of nurse-midwives were obtained from Queen Elizabeth Hospital (QECH: 83.2%) and Zomba Central Hospital (ZCH: 83.1%,) as compared to Mzuzu and Kamuzu central hospitals (MCH: 78%, KCH: 73%).

As regards the work-experience of nurses and midwives, results from the current study revealed that both clinical work and research experience had an effect on some facets of EBP. For clinical work experience, it was particularly pronounced (*P* = 0.006) that nurse-midwives with less than 5 years of clinical experience had the highest scores in their nursing attitude (82.2%) as compared to those both with five to 10 years (76%), and greater than 10 years of clinical work experience (64%). Furthermore, the results also showed significant differences in nursing practice (*P* = 0.013) and overall EBP (*P* = 0.035) between RNMs with research experience (Practice: 60.8%, EBP: 73.4%) and those without it (Practice: 55.9%, EBP: 78.1%). The rest of the differences in EBP amongst RNMs were not statistically significant (*P* > 0.05) amongst nurse-midwives based on both their clinical work and research experiences.

Upon running correlation analysis, results in Table 4 showed that total EBP amongst nurse-midwives was most strongly correlated to their knowledge levels (*r* = 0.891, *P* < 0.001). Nursing practice had only a strong relationship with total EBP (*r* = 0.782) whereas a moderate relationship between total EBP and nursing and midwifery attitude (*r* = 0.539) was revealed. The influence of the different aspects of EBP (attitude, practice, and knowledge level) on each other was generally weak (0.487 – r – 0.293) though statistically significant (*P* < 0.001). As such, after further proceeding to carry out stepwise regression analysis, the model summary (Table 5) and ANOVA for the regression model 3 (Table 6) revealed that all the models were found to be statistically significant (*P* < 0.001). Using the model coefficients highlighted in Table 7, it was revealed that for every unit increase in the knowledge levels of RNMs a 0.57% increase in the total EBP is expected, assuming nursing and midwifery practice and attitude of the RNMs are constant. Furthermore, total EBP for every unit increase in both nursing and midwifery attitude as well as practice amongst RNMs is also expected to increase.

**Table 4:** Pearson’s correlation matrix of Evidence-based practice (EBP) variables

Variables*	Total EBP	Practice	Attitude	Knowledge levels
Total EBP	**1**			
Practice	0.782	**1**		
Attitude	0.539	0.305	**1**	
Knowledge levels	0.891	0.487	0.293	**1**

*Correlations between all variables (%) are significantly different at *P* < 0.001

**Table 5:** Model summary for Step-wise multiple linear regression analysis

Model	R	R^**2**^	Adjusted R^**2**^	Std. Error of the Estimate	Change Statistics		
					R^**2**^ Change	F-Change	***p***-value (change)
1	.891^a^	0.794	0.793	6.436	0.794	696.782	0.000
2	.976^b^	0.953	0.952	3.082	0.159	609.239	0.000
3	0.999^c^	0.999	0.999	0.034	0.047	1,462,753.64	0.000

^a^The predictors for model 1 include: (Constant), score of knowledge levels (%)

^b^The predictors for model 2 include: (Constant), score of knowledge levels (%), score of nursing practice (%)

^c^The predictors for model 2 include: (Constant), score of knowledge levels (%), score of nursing practice (%), and score of nursing attitude (%)

**Table 6:** Analysis of Variance (ANOVA) summary for the regression model

Model^a^	Sum of Squares	df	Mean Square	F-statistic	***p***-value
Regression^b^	36,361.991	3	12,120.664	10,369,182.119	0.000
Residual	0.209	179	0.001		
Total	36,362.199	182			

^a^The dependent variable for the model is Total Evidence-based Practice score (%)

^b^The independent variable for the regression models include the (Constant), Practice, Attitude and Knowledge level scores (%)

**Table 7:** Coefficients used to determine the total score (%) for evidence-based nursing and midwifery practice (Total EBP)

Predictors	***β***	Std. Error	t-statistic	***p***-value	VIF^**a**^
(Constant)	−0.0138	0.0140	−0.983	0.327	
Practice	0.2499	0.0001	1948.043	0.000	1.36
Attitude	0.1668	0.0001	1209.443	0.000	1.14
Knowledge level	0.5834	0.0002	2996.279	0.000	1.35

^a^VIF is the variance inflation factor used to test for multicollinearity (values less than 5 are ideal, 5–10 are tolerable, and values greater than 10 represent a faulty model)

## Discussion of findings

To the best of our knowledge, this is the first study to describe RNMs knowledge, attitude and use of EBP among registered nurses and midwives in central hospitals in Malawi.

This study has shown that RNMs’ attitude towards EBP had the highest mean score followed by the knowledge or skills, and then practice. This shows that the RNMs viewed EBP positively and their attitude towards EBP seemed to be more positive than their knowledge or skills and use of EBP. This result is consistent with previous studies describing attitude, practices and knowledge or skills associated with EBP [1, 2, 35–37]. Over 80% of the respondents agreed that EBP was ‘fundamental to professional practice.’ They also reported ‘welcoming questions in own practice’ and ‘changing practice due to evidence found.’ These findings suggest that the nurses and midwives realise the importance of EBP and the need for implementing it. The positive attitude as Van Achterberg, Schoonhoven and Grol [32] observed allows for better strategies pertaining to EBP dissemination and implementation.

In this study, the RNMs showed that they had some knowledge about EBP. An overall mean score 5.46 ± 1.49 was received in the section of self-rated knowledge on EBP. However, knowledge was lacking relating to EBP skills, for instance, critical analysis of evidence against set standards (4.87 ± 1.52), retrieving evidence (4.77 ± 1.65), awareness of major information sources (4.57 ± 1.44%), information technology (4.53 ± 1.43), appropriate research skills (4.34 ± 1.53), and converting information needs into research questions (4.11 ± 1.56). As depicted in the results (Table 2) the RNMs scored high mean scores on identifying the research problem and sharing ideas and information with colleagues rather than the actual skills of obtaining the evidence. A similar response was also observed in previous studies [2, 9, 38–40]. Hansen and Severinsson [9] support the view in their study that EBP is the way to clinical decision-making. They emphasise the use of scientific research, sound judgment, and patient preference in respective contexts using various expertise. Brown et al. [2] also observed that although participants understood EBP as a new way of clinical decision-making, it required skills in its application to practice and also skills to research further on evidence that is relevant to the context of the investigation. It is important, therefore, that helping RNMs improve such aspects of their knowledge and/or skills in order to improve the overall EBP of RNMs in Malawian central hospitals. The central hospital managers should consider recruiting hospital librarians in their hospitals to promote EBP among RNMs and other staff. Where librarians have been used, they have introduced various programs on information literacy, importance of EBP, and implementation of EBP [41]. Nurse leaders, and nurse and midwife specialists should also intensify the use of performance appraisals so that specific needs of the nurses and midwives can be identified and applied during the continuing professional development programs.

The study also demonstrated significantly positive correlations (*P* < 0.001) between knowledge levels and nursing practice (*r* = 0.487) as well as the overall EBP (*r* = 0.782) of nurse-midwives. An improvement in the knowledge levels of RNMs could translate into their improved practice and thus again improve the overall EBP amongst RNMs in Malawian central hospitals. These results therefore demonstrate that EBP amongst RNMs can be improved most rapidly and significantly by improving primarily their knowledge levels, followed by improving their practical skills and lastly their attitude to the discipline. This explains the results presented in Table 3 which have shown that higher education qualifications are associated with high scores in knowledge levels amongst RNMs, whereas available work (research) experience is also associated with higher scores in nursing and midwifery practice. In the end, both educational qualification and research experience affect the overall EBP.

Female participants dominated the study (79.2%), a finding that is consistent with other international results especially in the nursing and midwifery professions where more females are reported than males [42]. The current study has revealed no statistically significant differences in RNMs’ practice, attitude, knowledge levels, and overall EBP based on gender (*P* > 0.05). Even though AbuRuz, Hayeah, Al-Dweik and Al-Akash [3] reported that female nurses conduct research less, have less positive and less knowledge about research compared to their male counterparts, Hasheesh and Ruz, [43] reported contrary results.

In this study, EBP amongst RNMs was not influenced by administrative roles or positions held by nurse-midwives in hospitals. This finding is consistent with a study conducted in Ethiopia [15]. This may be due to lack of managerial kills and EBP training. However, leadership is described as a key for creating an environment for generation and implementation of EBP [44]. Leaders have a responsibility to engage staff at all levels, support an EBP culture and allocate resources to provide the necessary infrastructure to promote clinical decision-making based on best available evidence [10]. These leaders should introduce (where necessary) and motivate the RNMs to participate in research activities like research trainings, scientific conferences and journal clubs to strengthen EBP in the clinical area. Nursing and midwifery leaders at the central hospitals need to understand the EBP process and be able to clearly articulate its meaning, use and impact on patient care [45]. It is time RNMs in this country had a shared vision and developed a model consisting of clinical bedside nurse-midwives and leaders at an individual hospital that can be used to standardise and support EBP.

This study has found that RNMs who had a master’s degree in nursing or midwifery tended to perceive fewer barriers to finding research compared to nurses-midwives with diplomas. This is consistent with recent studies [3, 37, 46, 47]. For instance, a study by Grant, Stuhmacher and Bonte-Eley [47], found that RNMs with master’s degrees often promoted EBP among their clinical nursing colleagues. Al Qadire [46] observed that teaching of EBP and its related skills are emphasised during postgraduate studies. This is supported by AbuRuz, Hayeah, Al-Dweik and Al-Akash [3] who indicated that the master’s degree curricula often contains more specialised courses about nursing and midwifery research methodology than those below them. At master’s level, RNMs are required to find significant clinical problems, perform integrated literature reviews, read and implement research findings, critique previous research, write proposals and publish scientific papers. This preparation makes the students at master’s level apply the steps of research, and learn how they can effectively implement EBP. RNMs with Master qualifications should, therefore, take an upper hand in facilitating a culture of EBP in the clinical area. This suggests that RNMs who have diplomas or even bachelor degrees who have not received any research or EBP training need additional education. This could be done by initiating comprehensive in-service training programmes on EBP [48], and/or offering scholarships for those who wish to complete a bachelor’s or master’s degree [49]. There is need to incorporate a course on EBP into RNMs training programs in higher educational institutions to enable these cadres to integrate EBP into their work after graduation.

This study found that RNMs who acquired their current qualification within 5 years perceived fewer barriers to finding research than those who obtained their current qualifications more than 5 years ago. This is consistent with previous studies [45, 50]. This greater knowledge of EBP with novice RNMs, when compared to experienced RNMs, is most likely attributable to modern-day nursing curriculums that include EBP which most seasoned RNMs lack [45]. Majid, Foo, Luyt et al. [51] also found that when nurses graduate from higher levels, including the baccalaureate programme, they were more capable to benefit from EBP activities. These RNMs are exposed to research process before they graduate. The main purpose of EBP is to recognise, appraise and apply the best available research finding [36]. This is probably why RNMs who had research experience had better knowledge, attitude, and practice due to exposure. As Chen, Wu, Zhou et al. [37] observed, there is need to offer EBP training programs inform of workshops, conferences, and lectures to those who had not received EBP education in college for them to obtain knowledge and skills about EBP at the workplace.

Failure to “make time in a work schedule for research” in the attitude subscale was identified as the main barrier to using EBP among RNMs in Malawi. This study also concurs with several previous studies [1, 18, 34, 38]. In a study by Majid, Foo, Luyt et al. [51], more than half of study participants reported perceived lack of time at their workplaces as one of the priority barrier to accessing and reviewing literature. According to Cummings, Estabrooks, Midodzi, Wallin and Hayduk [52], a better understanding of the nursing practice environment is crucial to the understanding and development of interventions to advance EBP. Hospital management and policy decision-makers, therefore, should consider making adjustments to RNMs work schedules or recruiting more so that they have additional time to attend classes on conducting EBP, reviewing relevant literature and planning practice changes [30]. However, previous researchers argued that lack of knowledge and skills in EBP could be underlining aspects of lack of time [18, 29, 34]. Without proper knowledge and skills for critically analysing evidence against set standards, retrieving evidence, determining the validity of material and converting information needs into research questions scored very low in this study (refer Table 2) might be too time consuming. Clinical integrated teaching on evidence-based processes would improve the knowledge, skills, attitude, and practice on EBP. Capacity building of RNMs on EBP processes is required to increase their knowledge on EBP to reduce time for searching for EBP information. This calls for cooperation between academic faculty and clinical RNMs.

The results of this study also showed that RNMs practising at QECH and ZCH have higher levels of knowledge, attitude and practice on EBP compared to MCH and KCH. QECH is the biggest hospital in Malawi and better resourced in terms of human resource and medical equipment than any other central hospital in the country. Its proximity of Kamuzu University of Health Science’s constituent colleges namely College of Medicine and Kamuzu College of Nursing, and Malawi College of Health Sciences to the facility makes it a better teaching facility than the rest. Since the EBP means scores observed among the four care settings in this study were small, future EBP educational programs should target RNMs practicing in all the four settings including those in the academia for better results.

### Strengths and limitations of the study

The study has some strengths. First, the study had a response rate of 87.9% which made the results to be generalised to the study population. Secondly, the study used an adopted validated instrument. The instrument was developed in the UK [34] with the aim of reporting development and validation of a self-report measure of knowledge practice and attitude of EBP among nurses. Reliability was established using Cronbach Alpha. Validity was established using construct and discriminant validity. The tool has been extensively used previously in USA [2, 40].

However, this study needs to be considered in light of certain limitations. First, the study is cross-sectional in nature. As such, the ability to infer cause and effect conclusions between study variables was hindered. Secondly, this study was based on self-reported data, which may be less accurate than other forms of measurement due to an inherent bias. Participants may answer scale items in a socially desirable manner. Thirdly, the study was conducted with RNMs of public hospitals and cannot be generalised to non-governmental hospitals (i.e. private, Christian Health Association of Malawi (CHAM) facilities and other populations). Despite the mentioned limitations, this study provides some insight on knowledge, attitude and use of EBP among RNMs in Malawi.

## Conclusions

Our findings add support for continued efforts to increase nurse-midwives’ education and create opportunities for RNMs to participate in career advancement opportunities and research. Higher levels of education may empower RNMs to act as autonomous practitioners who advocate for evidence-based innovations, and allow a spirit of inquiry to flourish.

The RNMs in this study were lacking knowledge and skills of EBP like research skills or critically analysing evidence against set standards, retrieving evidence, determining the validity of material found, and converting information needs into research questions. Furthermore, emphasis was put on ‘the sharing of information with colleagues’ rather than ‘tracking evidence or critical appraisal of evidence’ under practices. The fact that RNMs indicated that they were not fully practicing EBP due to some challenges which included lack of time show that they had a positive attitude towards EBP. This study indicates that although they were knowledgeable about EBP, there is need for further development of their capacity to retrieve and critically appraise evidence so that they can advocate more on EBP to the junior nurses and midwives, colleagues as well as their management.

## Data Availability

The datasets used and/or analysed during the current study are available from the corresponding author on reasonable request. Email: kasekapaul2016@gmail.com

## Abbreviations

ANOVA: Analysis of variance
EBP: Evidence-based practice
EBPQ: Evidence-based practice Questionnaire
ICN: International Council of Nurses
KCH: Kamuzu Central Hospital
MCH: Mzuzu Central Hospital
NMCM: Nurses and Midwives Council of Malawi
QECH: Queen Elizabeth Central Hospital
RNM: Registered Nurse Midwife
SPSS: Statistical Package for Social Scientists
VIF: Variance Inflation Factor
ZCH: Zomba Central Hospital
